# Targeting the Respiratory Syncytial Virus N^0^-P Complex with Constrained α-Helical Peptides in Cells and Mice

**DOI:** 10.1128/AAC.00717-20

**Published:** 2020-09-21

**Authors:** Marie Galloux, Nadège Gsponer, Vanessa Gaillard, Brice Fenner, Thibaut Larcher, Marthe Vilotte, Julie Rivière, Charles-Adrien Richard, Jean-François Eléouët, Ronan Le Goffic, Joelle Mettier, Origène Nyanguile

**Affiliations:** a VIM, INRAE, Jouy-en-Josas, France; b INRA, UMR 703 APEX, Nantes, France; c Université Paris-Saclay, INRAE, AgroParisTech, GABI, Jouy-en-Josas, France; d Université Paris-Saclay, INRAE, AgroParisTech, MICALIS, Jouy-en-Josas, France; e HES-SO Valais-Wallis, Institute of Life Technologies, Sion, Switzerland

**Keywords:** N^0^-P complex, RSV resistance mutants, antiviral agents, inhibitors, phosphoprotein, respiratory syncytial virus, stapled peptides

## Abstract

Respiratory syncytial virus (RSV) is the main cause of severe respiratory infection in young children worldwide, and no therapies have been approved for the treatment of RSV infection. Data from recent clinical trials of fusion or L polymerase inhibitors for the treatment of RSV-infected patients revealed the emergence of escape mutants, highlighting the need for the discovery of inhibitors with novel mechanisms of action. Here we describe stapled peptides derived from the N terminus of the phosphoprotein (P) that act as replication inhibitors. We demonstrate that these peptides inhibit RSV replication *in vitro* and *in vivo* by preventing the formation of the N^0^-P complex. The present strategy provides a novel means of targeting RSV replication with constrained macrocyclic peptides or small molecules and is broadly applicable to other viruses of the *Mononegavirales* order.

## Abstract (precis)

Respiratory syncytial virus (RSV) is the main cause of severe respiratory infection in young children worldwide, and no therapies have been approved for the treatment of RSV infection. Data from recent clinical trials of fusion or L polymerase inhibitors for the treatment of RSV-infected patients revealed the emergence of escape mutants, highlighting the need for the discovery of inhibitors with novel mechanisms of action. Here we describe stapled peptides derived from the N terminus of the phosphoprotein (P) that act as replication inhibitors.

## INTRODUCTION

Respiratory syncytial virus (RSV) is very contagious and represents the main cause of severe acute respiratory tract illness in young children worldwide. In 2005, RSV caused almost 34 million cases of lower respiratory infections in children under 5 years of age, with 3 to 10% of them requiring hospitalization, accounting for 45% of the total child admissions (1). In the United States, it has been estimated that RSV is responsible for 86,000 child hospitalizations per year, with an estimated cost of $394 million (2). A recent study performed by Pneumonia Etiology Research for Child Health (PERCH) across 7 countries revealed that RSV was the reason for hospitalization for 31% of all children hospitalized with severe pneumonia (3). In addition, the virus is also increasingly recognized as an important pathogen in the elderly population as well as in bone marrow transplant recipients (4). The current standard of care consists of prophylactic treatment of at-risk infants with palivizumab (Synagis), a monoclonal antibody that is administered monthly as an injectable during the peak season of infection (typically, November to March in Europe and in the United States). However, its limited efficacy (approximately 50%) and high cost (€5,000 per treatment) limit its use to preterm infants with bronchopulmonary dysplasia and chronic respiratory disease and newborns with congenital heart disease (2). As a result, 60% of at-risk children remain untreated, and no efficient therapy is available to treat the adult population.

**FIG 1:**
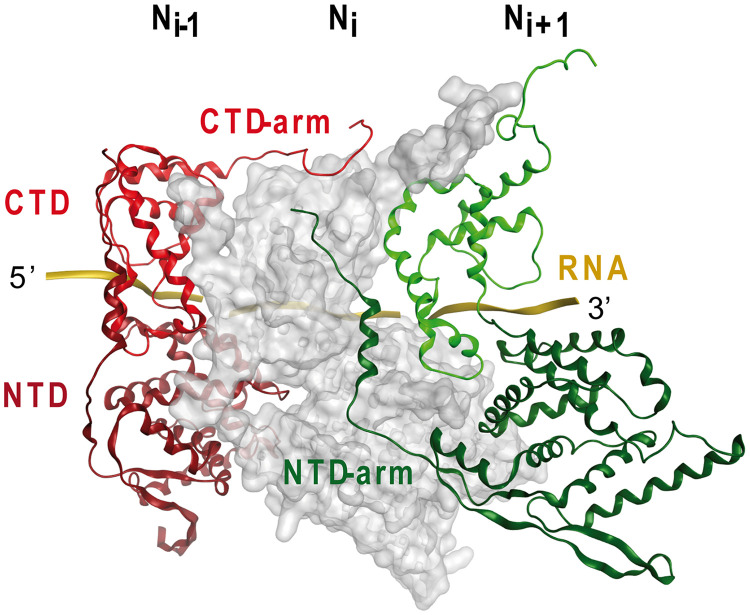
Partial representation of the nucleoprotein ring with 3 out of the 10 N subunits, showing the interactions between protomers (PDB accession number 2WJ8). The surface of the N*_i_* protomer is shown in gray. The ribbon structures of the N*_i_* _−_ _1_ and N*_i_* _+_ _1_ protomers are shown in red and green, respectively, and the RNA is shown in gold. The CTD and NTD are indicated on the structure of the N*_i_* _−_ _1_ protomer. The N*_i_* _−_ _1_ protomer CTD-arm, which binds atop the N*_i_* protomer CTD, and the N*_i_* _+_ _1_ protomer NTD-arm, which binds against the flank of the N*_i_* protomer, are shown.

RSV is an enveloped negative-strand RNA virus that belongs to the *Mononegavirales* order and that is a member of the *Pneumovirus* family (5). The genomic RNA of RSV is 15 kb in length and contains 10 tandemly linked genes that encode 11 proteins in the following order: the nonstructural NS1 and NS2, nucleoprotein (N), phosphoprotein (P), matrix (M), small hydrophobic (SH), glycoprotein (G), fusion (F), M2-1 and M2-2 (bicistronic), and large (L) proteins. To replicate, the negative-strand RNA genome must be converted into the antigenome positive strand by the RNA-dependent RNA L polymerase. The newly synthesized antigenome strand serves as the template for further copies of the genomic RNA. Both the neosynthesized antigenome and genome strands are encapsidated by nucleoprotein N to form ribonucleocapsids (RNCs). The nucleoprotein oligomerizes and enwraps the genomic RNA with its two globular domains, the N-terminal domain (NTD) and the C-terminal domain (CTD), linked through a hinge region (6). The NTD and the CTD have N- and C-terminal extensions, named NTD-arm and CTD-arm, respectively (Fig. 1), that play a key role in the formation of the nucleocapsid: the N*_i_* _−_ _1_ protomer CTD-arm binds atop of the N*_i_* protomer CTD, and simultaneously, the N*_i_* _+_ _1_ protomer NTD-arm binds against the flank of the N*_i_* protomer (*i* is the middle subunit of three adjacent N protomers). These two protein-protein interactions mediated by the CTD- and NTD-arms hold the protomers together and are therefore critical for the oligomerization of N. Prior to the encapsidation of genomic RNA, N is kept in an assembly-competent form known as N^0^ by binding to the chaperone phosphoprotein (P) until it is delivered to nascent synthesized viral RNA (4).

Because of the high propensity of nucleoprotein N to interact with RNA and oligomerize, the isolation and characterization of the N^0^-P complex are challenging and have not yet been successfully achieved, and the molecular mechanisms involved in the transition from the N^0^-P complex to the N-RNA nucleocapsid still remain to be elucidated. Bioinformatic studies have suggested that all P proteins of the *Mononegavirales* use their N-terminal subdomain (P N-ter) as a means to maintain N in its monomeric RNA-free N^0^ form (7). The resolution of the N^0^-P X-ray structures of Nipah virus (8), measles virus (MeV) (9), human metapneumovirus (HMPV) (10), Ebola virus (11), Marburg virus (12), vesicular stomatitis virus (VSV) (13), and parainfluenza virus 5 (PIV5) (14) confirmed this hypothesis and brought evidence that these viruses share a similar mechanism. In all cases, P N-ter competes with the binding of the N*_i_* _+_ _1_ protomer NTD-arm to the N*_i_* protomer, thereby preventing the oligomerization of N. However, the mechanism to prevent the binding of RNA appears to differ among viruses; in the case of VSV, P N-ter folds into a long α-helix and directly blocks the RNA-binding groove (13), while for MeV, HMPV, PIV5, and Nipah, Ebola, and Marburg viruses, P N-ter folds into two shorter α-helices and uses an allosteric mechanism to keep N^0^ in an open, RNA-free conformation. For RSV, although no X-ray structure of the N^0^-P complex has been resolved to date, recent studies have provided hints about how this complex might be structured. First, nuclear magnetic resonance (NMR) studies have identified a transient α-helix at the P N-ter spanning residues Asp12 to Ile24 (15). Second, the residues of P N-ter that are required to bind specifically to N^0^ have been identified by Ala scan mutagenesis, and it was shown that overexpression of P N-ter from residues 1 to 29 [P(1–29)] can impair RSV polymerase activity (16). Third, a biochemical study of the RSV N^0^-P complex in solution suggested a strong structural homology with the N^0^-P complex of HMPV (17). The X-ray structure of the HMPV N^0^-P complex revealed that P uses two structural motifs to bind to the N^0^ CTD subdomain: P(14–28) folds as an α-helix and binds atop the CTD, and P(1–12) is unfolded in an extended conformation and binds at the flank of the CTD (10). Superimposition of the N^0^-P complex with the N-RNA complex showed that the P peptide competes with the binding of the N*_i_* _+_ _1_ protomer NTD-arm and the N*_i_* _−_ _1_ protomer CTD-arm.

In the present work, we wished to investigate whether peptides derived from RSV P(1–30) can be used to inhibit RSV replication through preventing the oligomerization of N. Such a strategy has already been proposed for RSV and rabies and Nipah viruses (8, 16–18). However, developing peptides into a drug can be very challenging due to their poor bioavailability. Recently, the stapled peptide technology has emerged as a promising tool to solve this issue (19, 20). Nonnatural olefinic amino acids are incorporated into the peptides, and the olefinic side chains are cross-linked by ruthenium-catalyzed metathesis. The nature of the staple incorporated can increase dramatically the potency, proteolytic stability, and cellular permeation of the peptide, as it consists of a large hydrophobic all-hydrocarbon macrocycle (21). In the study described here, we performed a stapled peptide scan of the RSV P N-ter and we identified a peptide capable of inhibiting RSV infection *in vivo*.

## RESULTS

### Stapled peptide scan across the P N-terminal domain.

**FIG 2:**
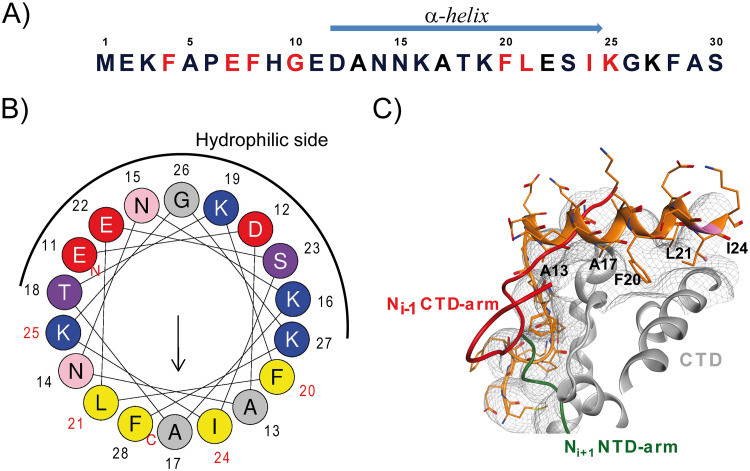
Primary amino acid sequence of the P N terminus and model of the P binding site on monomeric N. (A) The sequence of the first 30 N-terminal amino acid of P is presented, with residues identified to be critical for the interaction with N^0^ indicated in red. The location of the α-helix, characterized by NMR (15), is indicated by a blue arrow above the sequence. (B) Helical wheel representation (made with HeliQuest online software) of the putative α-helix located between residues 11 and 28 of P. Residues critical for N^0^ binding (16) are numbered in red. The hydrophilic face is indicated by a black half circle. Positively charged residues are in blue, negatively charged residues are in red, neutral residues are in gray, serine and threonine are in purple, uncharged residues are in pink, and hydrophobic residues are in yellow. (C) Model of the N^0^-P binding site. The surface of N^0^ is presented in gray. The ribbon structures of the peptide P(1–28) (orange), of the N*_i_* _−_ _1_ protomer CTD-arm (red), and of the N*_i_* _+_ _1_ protomer NTD-arm (green) are superimposed on the N surface, showing that the P binding site overlaps both the NTD- and CTD-arms of adjacent protomers. The putative P residues making hydrophobic contacts with N^0^ are shown on the α-helix in black. This model was generated with the Molecular Operating Environment (MOE) using the structures with PDB accession numbers 2WJ8 and 5FVD.

Previous NMR studies have shown that P(12–24) folds into an α-helix upon binding to N^0^ (15). The helical wheel representation of P(11–28) shows that the transient helix is composed of a core of hydrophobic residues on one face of the α-helix and hydrophilic residues on the other face (Fig. 2A and B), a characteristic of amphipathic helices. The amino acids that were identified by Ala scan mutagenesis to be required for the binding of P to N^0^ (16) are located on the predicted hydrophobic face of the helix. Consistent with this, modeling of the RSV N^0^-P complex revealed that Ala13, Gln14, Ala17, Phe20, Leu21, and Ile24 make van der Waals contact in the CTD N^0^ binding groove (17) (Fig. 2C). Based on these predictions, we decided to stabilize the transient α-helix of P(12–24). Given that at least 16 to 18 residues are usually required to successfully stabilize an α-helix in aqueous solution (22) and that Phe28 appeared to fit well into the helical wheel representation, we decided to focus on P(11–30). A stapled peptide scan of P(11–30) was performed by inserting the all-hydrocarbon cross-link at the hydrophilic face of the helix, the presumed noninteracting face of the helix. (19). We tested all *i*, *i* + 3 and *i*, *i* + 4 staples, which span one turn of the α-helix, as well as the *i*, *i* + 7 staples, which span two turns of the α-helix (*i* is the position at the N terminus of the peptide where the first amino acid used for stapling is incorporated) (Table 1). We also prepared negative controls, peptides 1d and 2d, where the staple was introduced at two positions, which should impair the binding of the peptide to N^0^. The peptides were synthesized by solid-phase peptide synthesis, and the staples were incorporated by closure of the macrocyclic bridge using ruthenium-mediated ring-closing olefin metathesis (19).

**TABLE 1:** Amino acid sequences, α-helical content, and IC_50_ values of single stapled peptides derived from P(11–30)^*a*^

Staple and peptide^*b*^	Sequence at position:																				% helicity	IC_50_ (μM)
	11				15					20					25					30		
Wild type	E	D	**A**	**N**	N	K	**A**	T	K	**F**	**L**	E	S	**I**	K	G	K	F	A	S	NA	7.9 ± 1.8
*i*; *i* + 3																						
1a	-	-	-	-	+	-	-	X	-	-	-	-	-	-	-	-	-	-	-	-	5.5	>100
1b		-	-	-	-	+	-	-	X	-	-	-	-	-	-	-	-	-	-	-	11.5	33.7 ± 2.5
1c	-	-	-	-	-	-	-	-	+	-	-	X	-	-	-	-	-	-	-	-	58.9	49.7 ± 26.1
** 1d**	-	-	-	-	-	-	-	-	-	-	+	-	-	X	-	-	-	-	-	-	24.2	>100
1e	-	-	-	-	-	-	-	-	-	-	-	-	+	-	-	X	-	-	-	-	30.7	37.6
1f	-	-	-	-	-	-	-	-	-	-	-	-	-	-	-	+	-	-	X	-	>100	1.3 ± 1.1
1g	-	-	-	-	-	-	-	-	-	-	-	-	-	-	-	-	+	-	-	X	25.5	>100
*i*; *i* + 4																						
2a	-	-	-	-	X	-	-	-	X	-	-	-	-	-	-	-	-	-	-	-	17.9	23.2 ± 7.4
2b	-	-	-	-	-	-	-	X	-	-	-	X	-	-	-	-	-	-	-	-	31.0	11.4 ± 5.3
2c	-	-	-	-	-	-	-	-	X	-	-	-	X	-	-	-	-	-	-	-	16.8	8.8 ± 1.8
**2d**	-	-	-	-	-	-	-	-	-	X	-	-	-	X	-	-	-	-	-	-	10.1	>100
2e	-	-	-	-	-	-	-	-	-	-	-	X	-	-	-	X	-	-	-	-	>100	13.6 ± 2.4
2f	-	-	-	-	-	-	-	-	-	-	-	-	X	-	-	-	X	-	-	-	46.5	NA
2g	-	-	-	-	-	-	-	-	-	-	-	-	-	-	-	X	-	-	-	X	24.1	ND
*i*; *i* + 7																						
3a	-	-	-	-	8	-	-	-	-	-	-	X	-	-	-	-	-	-	-	-	64.6	2.1 ± 0.7
3b	-	-	-	-	-	8	-	-	-	-	-	-	X	-	-	-	-	-	-	-	36.5	NA
3c	-	-	-	-	-	-	-	-	8	-	-	-	-	-	-	X	-	-	-	-	96.0	39.2 ± 640
3d	-	-	-	-	-	-	-	-	-	-	-	8	-	-	-	-	-	-	X	-	35.3	2.3 ± 1.1
3e	-	-	-	-	-	-	-	-	-	-	-	-	8	-	-	-	-	-	-	X	19.9	25.13

a 8, *R*-octenylalanine; X, *S*-pentenylalanine; +, *R*-pentenylalanine; -, no change in sequence from the wild type; IC_50_, half-maximal inhibitory concentration; NA, not applicable (the labeled probe displays a polarization signal higher than that of the N^mono^/labeled probe complex in the presence of the stapled peptide); ND, not determined. The key amino acids that were shown to be required to bind to N^0^ are underlined (16); the amino acids in bold are predicted with our model (Fig. 2C) to make hydrophobic contact with N^0^.

b Peptides 1d and 2d were used as negative controls and are identified in boldface.

**FIG 3:**
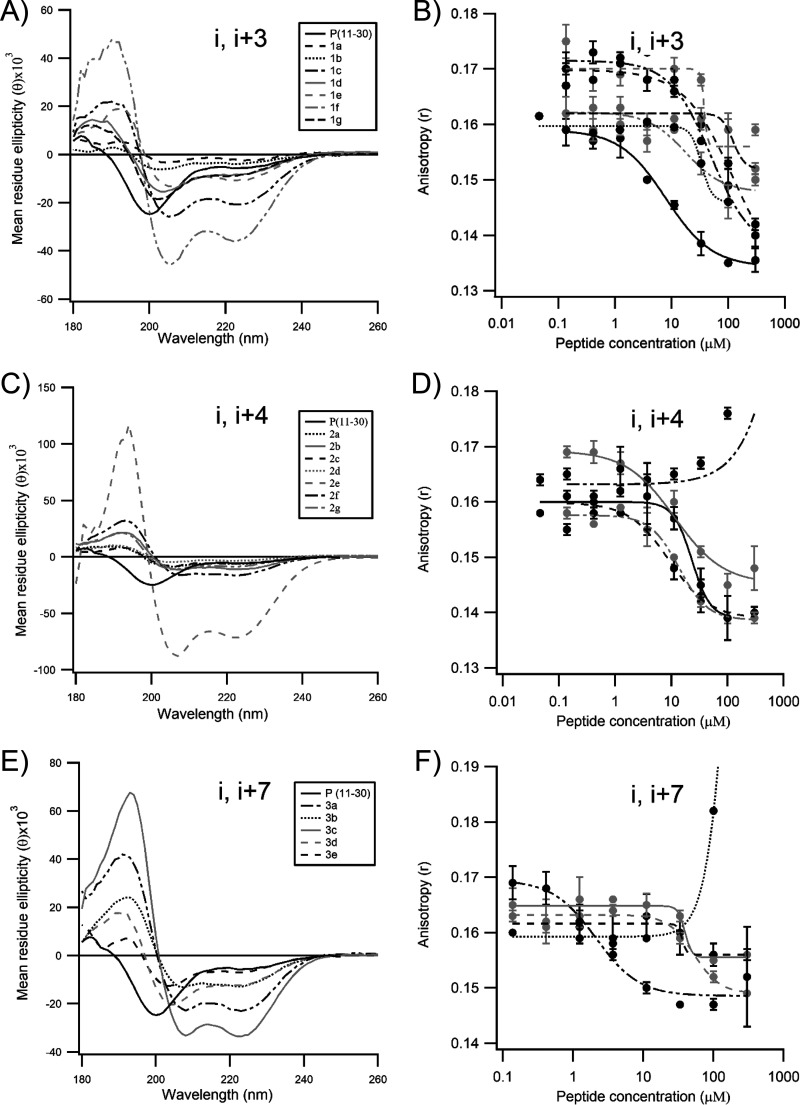
Biochemical characterization of the stapled peptides derived from P(11–30) tested in this study. The circular dichroism spectra (A, B, and C) and the results of the N^mono^ fluorescence polarization competition assay (D, E, and F) for peptides containing staples at positions *i*, *i* + 3; *i*, *i* + 4, and *i*, *i* + 7, respectively, are shown. Peptides 1d and 2d, in which the staple was introduced at two positions which should impair the binding of the peptide to N^0^, were used as negative controls. The curves were fitted in Igor Pro software using the Hill equation function. Error bars are standard deviations from duplicates.

To investigate whether the insertion of the staples in the P peptide resulted in an increase in the α-helical content, the peptides were analyzed by circular dichroism (CD). As it can be seen in Fig. 3, the CD spectra of wild-type peptide P(11–30) displayed a negative Cotton effect at approximately 195 nm, the signature of a random coil conformation. As expected, the insertion of a staple in the peptide resulted in a significant shift in the CD spectra, which then displayed two negative Cotton effects at approximately 207 and 222 nm, as well as a positive Cotton effect in the far-UV range, the CD signature of α helices (Fig. 3A to C). The stabilization of P(11–30) was observed in all staples tested, although the degree of α-helical content varied dramatically depending on the staple (5% to 100%) (Fig. 3 and Table 1). Peptides displaying α-helical contents higher than 30% were peptides 1c, 1e, and 1f for the *i*, *i* + 3 staples, peptides 2b, 2e, and 2f for the *i*, *i* + 4 staples, and peptides 3a, 3b, 3c, and 3d for the *i*, *i* + 7 staples. The α-helical stabilization appeared to be higher for the *i*, *i* + 7 staples overall. Altogether, these data are consistent with those from previous studies (15–17) and suggest that P N-ter does contain a transient α-helix subdomain.

### Affinity of stapled peptides for the monomeric N protein.

Next, we implemented a biochemical fluorescent polarization (FP) assay to investigate the impact of the incorporation of the staple on the binding affinity of the P peptides to N^0^. For this assay, fluorescently labeled P(1–40) was synthesized and incubated with monomeric N protein (N^mono^), a recombinant N^K170A/R185A^ double mutant reported previously (16). The K179A/R185A mutations were shown to impair the binding of RNA to the N protein (here named N^mono^), thereby enabling the purification of a monomeric, RNA-free RSV N protein. Upon binding to N^mono^, the rotation of the fluorophore of 6-carboxyfluorescein (FAM)–P(1-40) is reduced, thereby causing a significant increase in the polarization of the fluorescence signal. The difference in the fluorescent signal between the bound probe and the free probe could then be used to perform a dose-response competition assay in the presence of a competitor capable of displacing the labeled probe. The wild-type unstapled peptide, P(11–30), was able to displace the binding of the labeled probe to N^mono^ with a half-maximal inhibitory concentration (IC_50_) of 7.9 μM (Table 1). As expected, no competition could be observed for negative-control peptides 1d and 2d. In contrast to the beneficial effect observed in the circular dichroism studies, stapling did not improve the competitive binding activity of the studied peptides (IC_50_ value range, 1 to 50 μM). In two instances (peptides 1a and 1g), stapling appeared to be detrimental to the binding (Fig. 3D). Unexpectedly, we could not assess the competitive binding activity of peptides 2f and 3b in this assay, because the polarization signal increased instead of decreased in the presence of the competitor (Fig. 3E and F). We found that this artifact was due to the nonspecific binding of the peptides to the fluorescently labeled probe only, which most likely results in an oligomer inducing a higher polarization than the N^0^–P(1–40) bound complex (data not shown).

### Identification of stapled peptide inhibitors of RSV replication.

**FIG 4:**
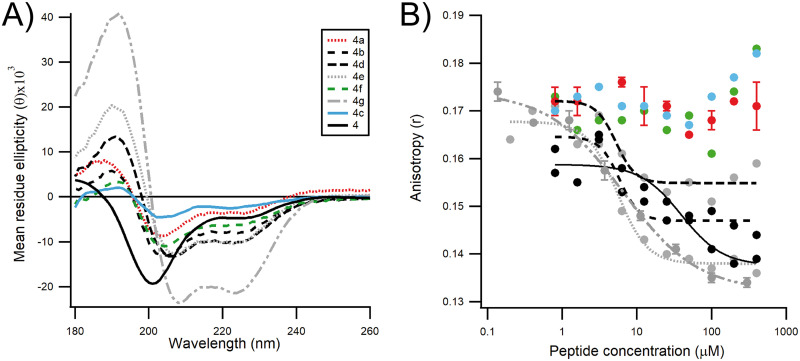
Effect of extending the single stapled peptides at the N terminus of P(7–30). (A) The circular dichroism spectra of single stapled peptides show that insertion of a staple at the N terminus does not stabilize the α-helical conformation. Peptide 4 without a staple was used as a negative control. (B) The results of the biochemical fluorescence competition assay of single stapled peptides show that the insertion of staples beyond P(12–25) does provide inhibitory activity to the peptides.

**FIG 5:**
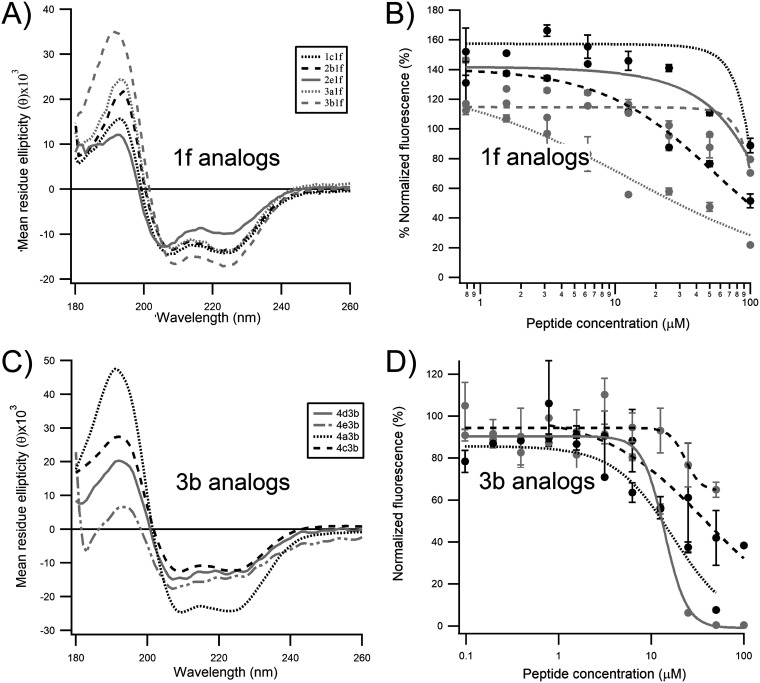
Effect of double-staple optimization on peptide 4. The single staples of P(7–30) were combined with the best staples of peptides 1f (A and C) and 3b (B and D) identified in the P(11–30) scan. (A, B) Circular dichroism spectra of double-stapled peptides derived from P(7–30). (C, D) Inhibition of RSV infection in HEp-2 cells by optimized double-stapled peptides. Cells were infected with rHRVS-mCherry at an MOI of 0.2, and the mCherry fluorescence was measured at 48 h postinfection. The curves were fitted in Igor Pro software using the Hill equation function. Error bars are standard deviations from duplicates. Data are representative of those from three independent experiments.

**FIG 6:**
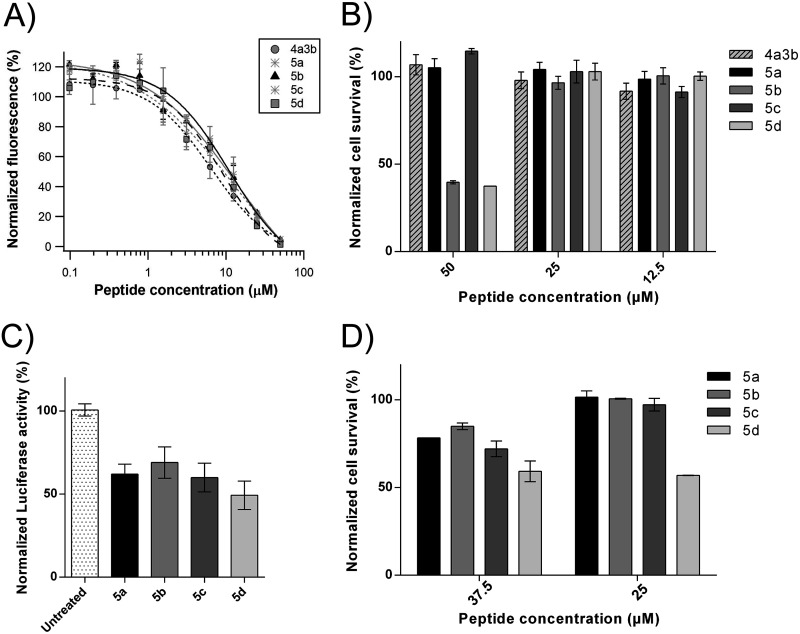
Validation of the antiviral activity and the specificity of double-stapled peptides derived from P(7–30) in cells. (A) Inhibition of RSV infection in HEp-2 cells by double-stapled peptides. Cells were infected with rHRVS-mCherry at an MOI of 0.2, and the mCherry fluorescence was measured at 48 h postinfection. The curves were fitted in Igor Pro software using the Hill equation function. Error bars are standard deviations from duplicates. Data are representative of those from three independent experiments. (B) HEp-2 cell viability upon treatment with peptides alone at peptide concentrations of 50, 25, and 12.5 μM. Error bars are standard deviations from duplicates. Data are representative of those from two independent experiments. (C) Inhibition of RSV polymerase complex by doubled-stapled peptides. BSRT7/5 cells were transfected with plasmids pP, pN, pM2-1, pL, and pM/Luc, together with pRSV-β-Gal for transfection standardization. At 6 h posttransfection, the medium was replaced by DMEM without antibiotics containing stapled peptides at 25 μM. The luciferase activities were quantified with an Infinite 200 Pro microplate reader and normalized based on β-galactosidase (β-Gal) expression and on the signal obtained for untreated cells. Error bars are standard deviations from triplicates. Data are representative of those from three independent experiments. (D) Viability of BRST7/5 cells transfected with the minigenome and treated with peptides at a concentration of 37.7 or 25 μM. Error bars are standard deviations from duplicates. Data are representative of those from two independent experiments.

To investigate if the P α-helix can be further stabilized on the N terminus, we extended the peptide length to P(7–30) and performed another stapled peptide scan at the N terminus of the peptide (Table 2). P(1–6) was not included because of the presence of Pro6, a strong helix-breaking residue (23) that most likely results in a random coil region at the N terminus of P. Similar to what had been observed with the stapled peptide scan of P(11–30), we measured an increase in the helical content in the range of from 17% to 48%, except for peptide 4, peptide 4a, and peptide 4c, which were mostly random coils (Fig. 4A). This is expected, since peptide 4 is unstapled and since peptide 4a and peptide 4c bear a staple beyond the region predicted to be a transient helix (15). In parallel, the binding affinity of the P peptides to N^0^ was assessed as described above using our FP biochemical binding assay (Fig. 4B). The wild-type unstapled peptide 4 was able to displace the binding of the labeled probe to N^mono^ with an IC_50_ of 39.4 μM (Table 2). Peptides 4a, 4c, and 4f were not able to compete with the binding of the labeled probe to N^0^ at peptide concentrations as high as 400 μM. However, the other peptides, peptides 4b, 4d, 4e, and 4g, showed IC_50_ values close to 5 to 6 μM (Table 2). To investigate whether double stapling can improve the inhibitory potency of such short peptides, as has been observed previously (24), we combined these staples with the best staples of peptides 1f and 3b found in the P(11–30) scan (Table 3). Circular dichroism analysis revealed that all double-stapled peptides contained a significant content of α-helical structure (30 to 75%), confirming that the insertion of a second staple further stabilized the secondary structure (Fig. 5A and C). However, these modifications did not improve the affinity for N^0^, as measured using the FP assay (Table 3). Nevertheless, we decided to assess the inhibitory activity of the resulting double-stapled peptides on RSV-infected cells. As the targeted N^0^-P complex is located in the cytoplasm, the antiviral activity depends both on the affinity of the peptides for N^0^ and on their capacity to enter the cell. It has been shown previously that the incorporation of the staples can result in enhanced cellular permeability through an endosomal uptake mechanism (20). To evaluate peptide antiviral activity, HEp-2 cells were infected with a recombinant RSV expressing the mCherry reporter gene, and the inhibitory activity of the stapled peptides was quantified by fluorescence analysis, as previously described (24, 25) (Fig. 5B and D). We found that although it was inactive in the FP competitive binding assay, peptide 4a3b was the most potent inhibitor, with a half-maximal response inhibitory concentration (EC_50_) of 14.6 μM (Table 3), whereas peptides 4d3b, 3a1f, 4c3b, 2b1f, 2e1f, and 4e3b were somehow less active (EC_50_, 19.8 μM, 25.3 μM, 39.9 μM, 41.0 μM, 67.9 μM, and 73.2 μM, respectively). Similar results were obtained when the cells were treated during and after infection, showing that peptides do not target virus entry and that their activity depends on long-lasting events mainly dependent on their capacity to cross the cellular membrane (data not shown). Given that no cytotoxicity was observed in HEp-2 cells with a peptide concentration as high as 100 μM (data not shown), the question of how peptide 4a3b was still able to inhibit viral replication arose. We hypothesized that the loss of one ion pair in peptide 4a3b, resulting from the replacement of Asp12 and Lys16 by the unnatural amino acids used to insert the staple, had a negative impact on the binding kinetics toward recombinant N^mono^. Ion pairs are thought to be particularly important in stabilizing α-helix domains in solution (26). Charged residues (Lys, Arg, Glu, Asp) located across adjacent turns of the helix can have an important effect on the thermodynamic stability of the helix. To investigate this, we introduced novel ion pairs or moved existing ion pairs at the noninteracting face of the peptide by inserting Lys and/or Glu at the *i*, *i* + 3 or *i*, *i* + 4 positions (Table 4). When the resulting peptides (peptides 5a to 5d) were tested in the FP assay, we found that the binding to N^mono^ was restored for all peptides tested. Consistent with previous studies, the best helix former was obtained when two (*i* + 4)E,K ion pairs were inserted (peptide 5a) and the worse helix former was obtained when a reverse (*i* + 4)K,E bridge was inserted (peptide 5d) (26). For all peptides modified with these ion pairs, inhibition of viral infectivity was maintained at EC_50_ values similar to those for parent peptide 4a3b (Fig. 6A). However, peptides 5b and 5d showed some toxicity at a peptide concentration of 50 μM (Fig. 6B). Altogether, these data suggest that peptide 4a3b inhibits replication in HEp-2 cells by targeting the N^0^-P complex.

**TABLE 2:** Amino acid sequences, α-helical content, and IC_50_ values of single stapled peptides derived from P(7–30)^*a*^

Peptide	Sequence at position:																								% helicity	IC_50_ (μM)
	7			10					15					20					25					30		
4	**E**	**F**	H	**G**	E	D	**A**	**N**	N	K	**A**	T	K	**F**	**L**	E	S	**I**	K	G	K	F	A	S	NA	39.4 ± 16.7
4a	-	-	+	-	-	X	-	-	-	-	-	-	-	-	-	-	-	-	-	-	-	-	-	-	9.5	>400
4b	-	-	-	-	-	+	-	-	X	-	-	-	-	-	-	-	-	-	-	-	-	-	-	-	21.7	5.8 ± 1.1
4c	-	X	-	-	-	X	-	-	-	-	-	-	-	-	-	-	-	-	-	-	-	-	-	-	6.5	>100
4d	-	-	-	-	-	X	-	-	-	X	-	-	-	-	-	-	-	-	-	-	-	-	-	-	28.0	5.2 ± 1.1
4e	-	-	8	-	-	-	-	-	-	X	-	-	-	-	-	-	-	-	-	-	-	-	-	-	28.8	5.5 ± 0.8
4f	-	-	-	-	-	8	-	-	-	-	-	-	X	-	-	-	-	-	-	-	-	-	-	-	17.2	>100
4g	-	-	-	-	-	-	-	-	-	8	-	-	-	-	-	-	X	-	-	-	-	-	-	-	47.6	6.5 ± 1.7

a 8, *R*-octenylalanine; X, *S*-pentenylalanine; +, *R*-pentenylalanine; -, no change in sequence from the wild type; IC_50_, half-maximal inhibitory concentration; NA, not applicable. The key amino acids that were shown to be required to bind to N^0^ are underlined (16); the amino acids in bold are predicted with our model (Fig. 2C) to make hydrophobic contact with N^0^.

**TABLE 3:** Amino acid sequences, α-helical content, and IC_50_ and EC_50_ values of double-stapled peptides derived from P(7–30)^*a*^

Peptide	Sequence at position:																								% helicity	IC_50_ (μM)	EC_50_ (μM)
	7			10					15					20					25					30			
4	**E**	**F**	H	**G**	E	D	**A**	**N**	N	K	**A**	T	K	**F**	**L**	E	S	**I**	K	G	K	F	A	S	NA	39.4 ± 16.7	>100
1c1f	-	-	-	-	-	-	-	-	-	-	-	-	+	-	-	X	-	-	-	+	-	-	X	-	39.2	>100	>100
2b1f	-	-	-	-	-	-	-	-	-	-	-	X	-	-	-	X	-	-	-	+	-	-	X	-	37.6	NA	41.0 ± 0.5
2e1f	-	-	-	-	-	-	-	-	-	-	-	-	-	-	-	X	-	-	-	B	-	-	X	-	27.0	NA	67.9 ± 0.0
3a1f	-	-	-	-	-	-	-	-	8	-	-	-	-	-	-	X	-	-	-	+	-	-	X	-	37.7	NA	25.3 ± 17.9
3b1f	-	-	-	-	-	-	-	-	-	8	-	-	-	-	-	-	X	-	-	+	-	-	X	-	46.6	NA	>100
4a3b	-	-	+	-	-	X	-	-	-	8	-	-	-	-	-	-	X	-	-	-	-	-	-	-	73.2	>100	14.6 ± 13.9
4c3b	-	X	-	-	-	X	-	-	-	8	-	-	-	-	-	-	X	-	-	-	-	-	-	-	34.2	64.0 ± 1.7	39.9 ± 4.5
4d3b	-	-	-	-	-	X	-	-	-	B	-	-	-	-	-	-	/	-	-	-	-	-	-	-	36.1	19.8 ± 9.0	19.8 ± 9.0
4e3b	-	-	8	-	-	-	-	-	-	B	-	-	-	-	-	-	/	-	-	-	-	-	-	-	51.1	57.9 ± 21	73.2 ± 31.1

a 8, *R*-octenylalanine; X, *S*-pentenylalanine; +, R-pentenylalanine; /, *S*-octenylalanine; B, bis-*S*-pentenylalanine; -, no change in sequence from the wild type; IC_50_, half-maximal inhibitory concentration; EC_50_, half-maximal response inhibitory concentration; NA, not applicable (the labeled probe displays a polarization signal higher than that of the N^mono^/labeled probe complex in the presence of the stapled peptide). The key amino acids that were shown to be required to bind to N^0^ are underlined (16); the amino acids in bold are predicted with our model (Fig. 2C) to make hydrophobic contact with N^0^.

**TABLE 4:** Amino acid sequences, α-helical content, and IC_50_ and EC_50_ values of double-stapled peptides derived from peptide 4a3b^*a*^

Peptide	Sequence at position:																								% helicity	IC_50_ (μM)	EC_50_ (μM)
	7			10					15					20					25					30			
4a3b	**E**	**F**	+	**G**	E	X	**A**	**N**	N	8	**A**	T	K	**F**	**L**	E	X	**I**	**K**	G	K	F	A	S	73.2	>100	14.6 ± 13.9
5a	-	-	-	-	-	-	-	-	E	-	-	-	-	-	-	-	-	-	-	K	-	-	-	-	>100	0.83 ± 0.13	18.4 ± 9.3
5b	-	-	-	-	-	-	-	-	E	-	-	-	-	-	-	A	-	-	-	-	-	-	-	-	>100	1.94 ± 0.26	16.9 ± 5.1
5c	-	-	-	-	-	-	-	-	-	-	-	-	E	-	-	K	-	-	-	-	-	-	-	-	79.7	1.84 ± 0.18	18.5 ± 10.6
5d	-	-	-	-	K	-	-	-	E	-	-	-	-	-	-	-	-	-	-	-	-	-	-	-	75.7	14.21 ± 2.94	6.0 ± 4.2

a 8, *R*-octenylalanine; X, *S*-pentenylalanine; +, *R*-pentenylalanine; -, no change in sequence from the wild type; IC_50_, half-maximal inhibitory concentration; EC_50_, half-maximal response inhibitory concentration. The key amino acids that were shown to be required to bind to N^0^ are underlined (16); the amino acids in bold are predicted with our model (Fig. 2C) to make hydrophobic contact with N^0^.

To confirm that the double-stapled peptides identified here (peptides 5a to 5d) specifically target the N^0^-P complex, we tested the inhibitory activity of the peptides using a plasmid-based RSV minigenome replication assay (27). Briefly, the dicistronic RSV minigenome pM/Luc was cotransfected into BSRT7/5 cells expressing T7 RNA polymerase, together with plasmids pN, pP, pL, and pM2-1, resulting in the replication and transcription of the minigenome. Hence, preventing the formation of an N^0^-P complex competent for genomic or antigenomic RNA encapsidation would result in a decrease in the production of the luciferase (Luc) reporter. As shown in Fig. 6C, incubation of the cells in the presence of 25 μM peptides reduced approximately 30% to 40% of the luciferase activity compared to that of the control untreated cells. It is noteworthy that at this concentration no cellular toxicity was detected for any peptide except peptide 5d, which induced 40% cell mortality at this concentration (Fig. 6D). Given that no toxicity for uninfected HEp-2 cells was seen in the viral cellular inhibition assay with 50 μM peptides 5a and 5c (Fig. 6B), the toxicity observed here at concentrations higher than 30 μM was most likely due to the transfection of the plasmids with the Lipofectamine reagent and not to the administration of the peptides. Previously, we have shown, using the same minigenome assay, that the replacement of P residues critical for the interaction with N^0^ by Ala resulted in a 50% reduction in polymerase activity (16). We have also shown that the overexpression of P(1–29) led to the partial inhibition of the polymerase activity. The data obtained with the stapled peptides 5a, 5b, and 5c in the present study are consistent with these previous observations.

### *In vivo* activity of peptide 5a in living mice.

**FIG 7:**
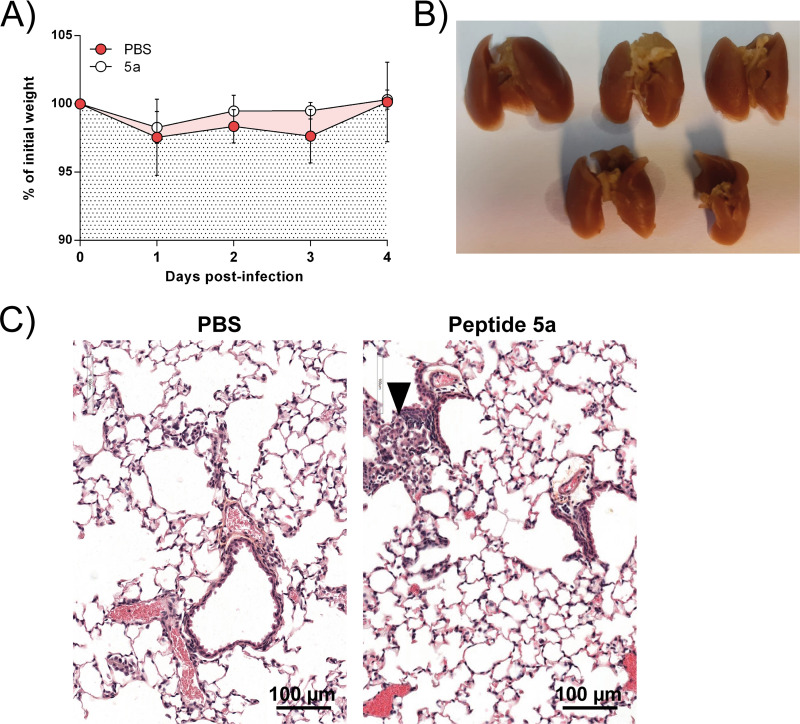
*In vivo* toxicity assessment of peptide 5a. Groups of 5 BALB/c mice were treated at days 0 and 2 either with 50 μl of PBS or with peptide 5a in PBS (50 μl at 215 μM) and sacrificed at day 4. (A) Daily body weight monitoring of BALB/c mice treated with or without peptide 5a. (B) View of the lungs of euthanized mice treated with peptide 5a. (C) Histopathological analysis of lungs treated with PBS or peptide 5a. Sections of fixed lungs were subjected to HES staining. Compared to control mice (left), animals treated with peptide 5a (right) showed small scattered foci of mixed inflammatory cell infiltration (arrowhead) in the lung parenchyma.

**FIG 8:**
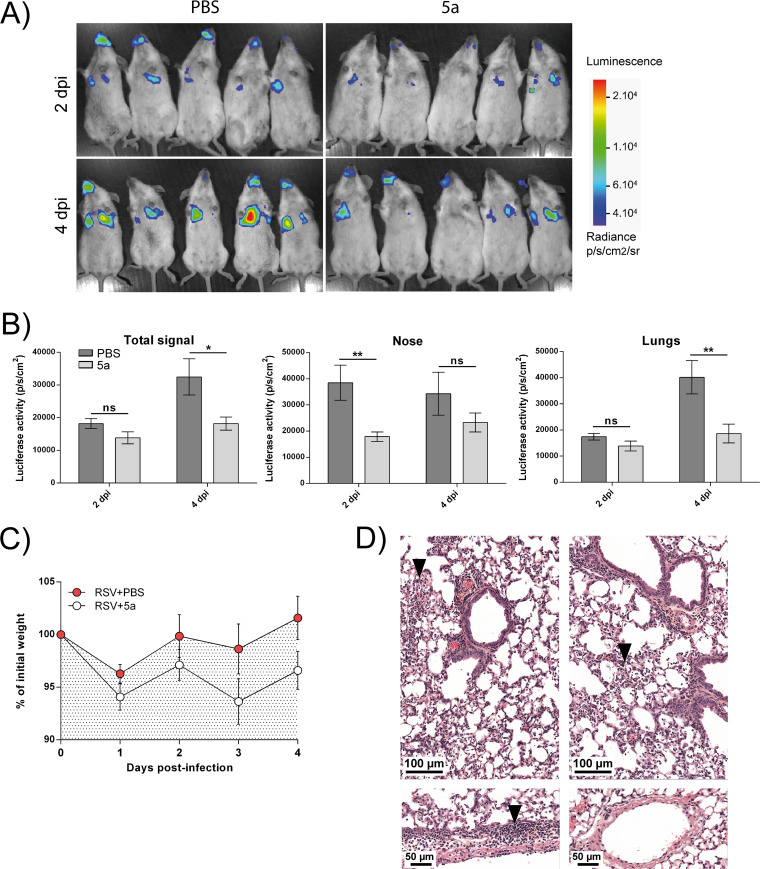
*In vivo* anti-RSV activity of stapled peptide 5a in mice. Groups of 5 female BALB/c mice were treated i.n. with peptide 5a or PBS as a control. The mice were then infected with 1 × 10^5^ PFU of rHRSV-Luc intranasally. The bioluminescence was measured at 2 dpi, and the mice were treated a second time. Finally, bioluminescence was measured at 4 dpi, the mice were sacrificed, and the lungs were collected. (A) Bioluminescence was measured by inoculation of d-luciferin i.n. (7 mg · kg^−1^) and by using an IVIS. The scale on the right indicates the average radiance (sum of the number of photons per second from each pixel inside the ROI/number of pixels). p/s/cm^2^/sr, number of photons per second per square centimeter per steradian. (B) Bioluminescence activities were quantified using Living Image software. The whole luminescent signal or specific signals in the nose or lungs of infected mice were quantified. The significance of the difference between groups was determined using the Mann-Whitney test (*, *P* < 0.05; **, *P* < 0.01; ns, not significant). p/s/cm^2^, number of photons per second per square centimeter. (C) Body weight monitoring of RSV-infected mice treated or not treated with peptide 5a. (D) Histopathological analysis of lungs of RSV-infected mice treated with PBS or with peptide 5a. Sections of fixed lungs were subjected to HES staining. Extensive suppurative pneumonia (top) was observed in all RSV-inoculated animals, with the alveolar presence of neutrophils and cellular debris indicated (arrowhead). Untreated animals additionally displayed some marked vascular lesions (bottom left). Treatment with peptide 5a reduced the intensity of pneumonia, and the occurrence of vascular lesions was noteworthy (bottom right). (Top and bottom) Representative lungs sections at low and high magnifications, respectively.

Next, we assessed the antiviral activity of our lead stapled peptide in a mouse RSV infection model (25). Given the similar range of potency that was observed for our peptide 5a to 5d series in the cellular viral and minigenome inhibition assays, peptide 5a was selected for this study, first, because it displayed the highest affinity toward the N^mono^ value in the biochemical inhibition assay (Table 4) and, second, because it showed no toxicity (Fig. 6B and D). We first investigated the potential toxicity of peptide 5a *in vivo* by treatment of BALB/c mice 8 weeks of age (*n* = 5) by intranasal (i.n.) administration of peptide 5a (50 μl at 215 μM in phosphate-buffered saline [PBS]) or PBS at days 0 and 2. Daily monitoring of the mouse body weight from days 0 to 4 did not reveal any signs of toxicity (Fig. 7A). At day 4, the mice were sacrificed and the lungs were collected to detect potential lesions. As shown in Fig. 7B, no macroscopic sign of toxicity was observed upon treatment with peptide 5a. Histological analysis of the lungs revealed some focal lesions of the pulmonary parenchyma with infiltration by neutrophils for 3 of the 5 animals treated with peptide 5a, in contrast to the findings for the untreated control mice (which were administered PBS), which showed no lesions (Fig. 7C). To test the antiviral activity of peptide 5a, BALB/c mice 8 weeks of age (*n* = 5) were treated as described above by intranasal administration of peptide 5a (50 μl at 215 μM in PBS) or PBS, followed by i.n. inoculation of a recombinant human RSV (rHRSV) encoding the gene for firefly luciferase, rHRSV-Luc (1 × 10^5^ PFU) (25). The mice were then anesthetized at 2 days postinjection (dpi), and viral replication was quantified using an *in vivo* imaging system (IVIS) after i.n. injection of d-luciferin. Although no significant difference was detected at this time point when considering the whole luminescence signal, a 2-fold reduction in the level of RSV replication was detected in the noses of mice treated with peptide 5a compared to that in the noses of untreated mice (Fig. 8A and B). Peptide 5a was then administered a second time to the mice, and replication was measured at 4 dpi. As shown in Fig. 8B, a significant reduction in the amount of bioluminescence was detected at 4 dpi in mice treated with peptide 5a compared to that detected in the untreated control group. Furthermore, the bioluminescence in the lungs was significantly reduced in mice treated with peptide 5a compared to that in the untreated RSV-infected mice. Daily monitoring of mouse body weights revealed that RSV infection did not induce any significant weight loss (Fig. 8C). This result is not surprising, since mice infected by RSV under experimental conditions similar to those used in the present study did not show clinical symptoms, as previously reported (25). Likewise, a statistically nonsignificant loss of weight was observed in infected animals which were treated with peptide 5a. Histological analysis of the lungs showed that RSV infection led to a multifocal extensive marked interstitial pneumonia, characterized by a diffuse thickening of the alveolar walls with mixed inflammatory cells. Mild periarteritis sometimes associated with focal hemorrhages and mild bronchitis with intraluminal necrotic debris were also observed in all 5 animals tested (Fig. 8D). In contrast, the intensity of interstitial pneumonia decreased upon inoculation of peptide 5a. Consistent with this observation, the vascular and bronchial lesions were less severe and were seen in only 2 out of 5 animals. These observations confirm the antiviral effect of peptide 5a.

Altogether, these data show that stapled peptides targeting the N^0^-P complex are capable of inhibiting RSV infection *in vivo*. Furthermore, no major sign of *in vivo* toxicity was observed in the present study.

## DISCUSSION

RSV is the main cause of severe respiratory infection in young children worldwide, and no therapies have been approved for the treatment of infections caused by RSV. Most of the antivirals under development aim at targeting the fusion protein (F), responsible for virus entry (28, 29), or the enzymatic activities of L polymerase (30–32). Importantly, most of the studies focusing on these antivirals report the appearance of escape mutants upon selection pressure (33–39). The detection of novel specific targets is thus warranted to support the development of combination therapies to minimize the emergence of resistance. Recent reports suggest that the RSV N^0^-P complex may be a suitable target for the development of antivirals. According to these studies, the N terminus of P (P N-ter) prevents the oligomerization of N, required for specific encapsidation of the viral antigenome and genome, through folding into an α-helix and binding in a region of N where the CTD- and NTD-arms of the N protomers interact (15–17). Consistent with these observations, the overexpression of P N-ter was shown to be inhibitory in an RSV minigenome replication assay (16). Here, we wished to develop peptide inhibitors derived from P N-ter using the stapled peptide technology (19), with the aim to stabilize the transient α-helical conformation of this P subdomain.

**FIG 9:**
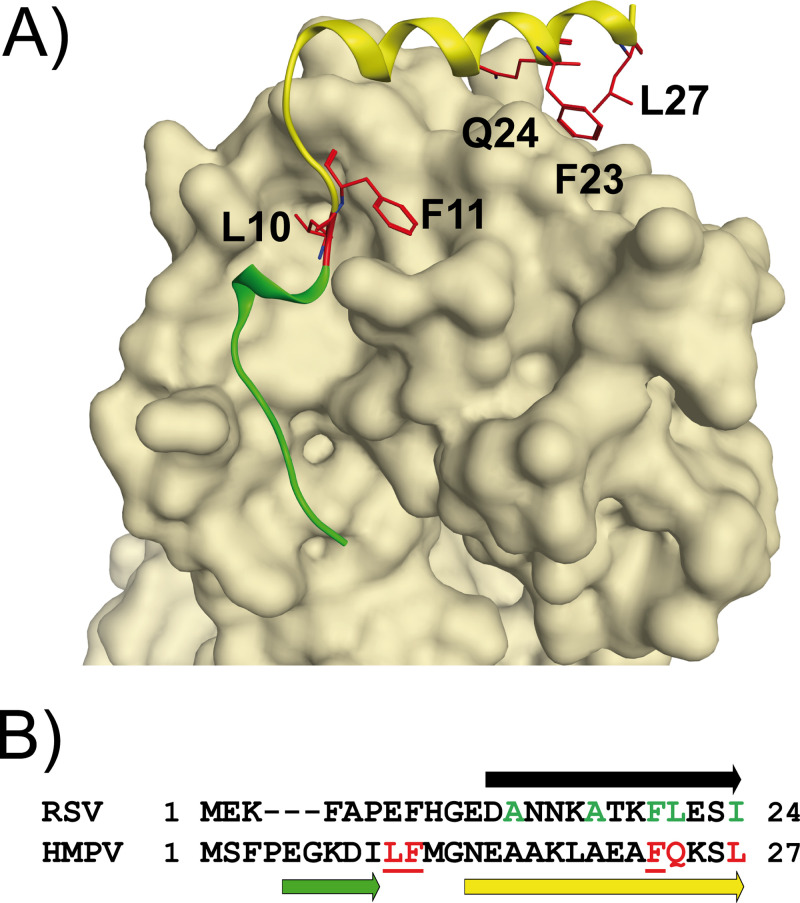
Comparison of the N terminus of the RSV and HMPV P proteins. (A) HMPV N^0^-P complex. The surface of N^0^ is shown in light brown. P(1–28) is shown as a ribbon. HMPV P binds to a flat surface atop the N^0^ CTD using a long α-helix (shown in yellow); it also binds at the flank of the N^0^ CTD into a big cavity using one turn of the α-helix and a random coil domain (shown in green). The side chains of P residues that make strong hydrophobic contact with N^0^ are highlighted in red. (B) Primary sequence alignment of the RSV and HMPV P N-ter motifs (7). The putative α-helix of RSV P is shown as a black arrow above the sequence. The RSV P residues making hydrophobic contact with N^0^ are highlighted in green. The long and short α-helices of HMPV P N-ter are represented as yellow and green arrows below the sequence, respectively. The key residues binding to N^0^ are color coded in red.

Using this chemistry, we performed a stapled peptide scan of sequences derived from P N-ter. We observed that the insertion of a staple at the noninteracting face of the predicted α-helix of P stabilizes the α-helical configuration. We identified novel double-stapled peptides that interfere with the binding of N^0^ to P, thereby preventing the delivery of N to the nascent RNA, unlike the unstapled peptides, which were previously shown to not be capable of inhibiting RSV in cells (40). Of all the single staples that were screened, only the staples inserted in P(15–30) could successfully stabilize the α-helix, corroborating the hypothesis that the α-helix must be located within Asp12 and Lys25 (15, 16). Our efforts to further stabilize the helix at the N terminus of P(7–12) were unsuccessful. Given that the N terminus contains an α-helix-breaking residue (Pro6), it is therefore unlikely that P(6–12) folds into a second short α-helix, as it has been described for other viruses (8–12). This observation is consistent with the findings of the bioinformatics studies of Karlin and Belshaw (7), which predicted that all *Pneumovirinae* have a conserved N-terminal motif, named *mir*, located between Phe8 and Phe20 in RSV. Recently, the X-ray structure of the N^0^-P complex of HMPV has been solved (10), revealing that HMPV P uses two α-helices to contact N^0^, a long helix spanning Gly12 to Leu26 and a short helix spanning Glu5 to Ile9 (yellow and green, respectively, in Fig. 9A). These two helices bind to N^0^ in an L shape and are connected by Leu10 and Phe11, which make a deep hydrophobic contact with N^0^. The alignment of the RSV and HMPV P sequences shows a remarkable similarity between the long helix of HMPV and the predicted α-helix of RSV (Fig. 9B). The HMPV P residues making the key hydrophobic contact with N^0^, i.e., Phe23, Gln24, and Leu27, correspond to Phe20, Leu21, and Ile24, respectively, in RSV. These residues were identified to be critical in our former biochemical studies and appear to be conserved between RSV and HMPV (16). Strikingly, the two other amino acids making the hydrophobic contact with N^0^ in HMPV P (Ala16 and Ala20) are also conserved in RSV (Ala13 and Ala17, respectively; Fig. 9B). Furthermore, the two HMPV P residues Leu11 and Phe12, which are deeply buried in the N^0^ cavity, correspond to Glu7 and Phe8, respectively, in RSV. As the Phe8Ala substitution was also found to result in a drop of polymerase activity using the minigenome assay, it is also possible that this residue makes an important hydrophobic contact with N^0^ (16). Altogether, these data strongly suggest that the binding of the predicted α-helix of RSV P(12–25) to N^0^ is similar to that of the long helix of HMPV P. Whether a short helix is also used by RSV, as is the case in HMPV, remains to be seen, but this seems to be unlikely, given the presence of a proline and charged polar residues at the N terminus of the sequence.

Of the most interest, peptide stapling was shown to facilitate the cellular uptake of our peptides, a condition necessary to successfully target the N^0^-P complex, since the mechanism of viral replication occurs within cells. We optimized stapled peptides derived from P(7–30) and were able to achieve inhibition of viral replication in cell culture with an EC_50_ of approximately 10 μM. Importantly, we found that the most active peptide, peptide 5a, inhibited viral replication *in vivo* in a mouse model. Usually, peptide antiviral strategies target the viral entry step because this mechanism has the advantage of being extracellular and therefore does not require a cell-permeant molecule to be inhibited. To prevent proteolytic degradation and improve the binding affinity to the target, peptides are constrained with various macrocyclization chemistries. A plethora of examples has been reported, including the insertion of a lactam bridge in RSV F (41, 42) or the influenza virus hemagglutinin stem (43) and the insertion of staples in RSV F (24, 44), HIV gp41 (21, 45), and Ebola virus GP2 (46). Because the mechanism for viral replication occurs intracellularly, cell-permeant molecules are needed to inhibit the N^0^-P complex. Despite the fact that the peptide was administered through nasal inhalation, peptide 5a was still capable of reaching the upper and lower respiratory tracts of living mice and inhibit viral replication. This is a significant achievement, suggesting that stapled peptides may be used to develop novel antivirals targeting the N^0^-P complex.

Of course, a medicinal chemistry effort is still needed to improve the potency of these molecules, such as the replacement of critical residues of P by natural and nonnatural amino acids with the aim to enhance the binding affinity of the stapled peptides for N^0^.

In conclusion, we have shown that constrained α-helical stapled peptides derived from P N-ter can inhibit RSV replication by targeting the N^0^-P complex. Based on the structural homology between the N^0^-P complexes of the *Mononegavirales*, such a strategy could also be used for all viruses of this order. More specifically, given the strong sequence homology between the N termini of the RSV and HMPV P proteins, it can be expected that the peptides identified in the work presented here may be directly amenable to the inhibition of HMPV. Regardless of whether stapled peptides, other macrocyclic peptides, or small molecules are used to target the N^0^-P interaction, the present strategy is a novel means to develop antivirals with activity against RSV and/or other *Mononegavirales* and opens new perspectives for combination therapies.

## MATERIALS AND METHODS

### Materials.

9-Fluorenylmethoxy carbonyl (Fmoc)-amino acids and coupling reagents were purchased from Aapptec, Bachem, PolyPeptide, and Sigma-Aldrich. The nonnatural olefin-containing amino acids were purchased from Okeanos Technology Co., Ltd. Solvents were purchased from Acros Organic, Biosolve, and Sigma-Aldrich.

### Cell culture.

HEp-2 cells (ATCC number CCL-23) were maintained in Eagle’s minimum essential medium (EMEM) supplemented with 10% fetal calf serum (FCS), 2 mM l-glutamine, and penicillin-streptomycin solution. BHK-21 cells (clone BSRT7/5) constitutively expressing the T7 RNA polymerase (47) were grown in Dulbecco modified essential medium (DMEM) supplemented with 10% FCS, 2 mM glutamine, and antibiotics. Cells were grown in an incubator at 37°C in 5% CO_2_. Recombinant human RSV strains corresponding to the RSV Long strain expressing either the mCherry or the luciferase protein (rHRSV-mCherry and rHRSV-Luc, respectively) were amplified and titrated as previously described (25). All experiments with RSV were carried out in biosafety level 2 facilities.

### Peptide synthesis.

Peptides were synthesized by solid-phase peptide chemistry on Rink Amide AM resin LL (100 to 200 mesh; Novabiochem), using an Apex 396 automated multiple-peptide synthesizer (Aapptec) at a 50-μmol scale. Each coupling was performed for 1 h at room temperature, using 200 μmol of Fmoc-amino acid preactivated with 190 μmol of 1H-benzotriazolium-1-[bis(dimethylamino)methylene]-5-chloro-hexafluoro-phosphate-(1-),3-oxide (HCTU) and 400 μmol of diisopropyldiethylamine (DIEA) in *N*-methyl-2-pyrrolidone (NMP). For the coupling following the nonnatural olefinic amino acids, HCTU was replaced by 190 μmol of 1-[bis(dimethylamino)methylene]-1H-1,2,3-triazolo[4,5-b]pyridinium 3-oxid hexafluorophosphate (HATU), and the coupling was performed twice for 1 h each time at room temperature. Following final Fmoc deprotection and N-terminal acetylation, the metathesis was performed under constant nitrogen degassing in a 2-ml solution containing 10 mM 1st-generation Grubb’s catalyst in dichloroethane (DCE). The metathesis was performed for 2 h at room temperature. The peptides were deprotected and cleaved from the resin with a cleavage cocktail consisting of trifluoroacetic acid-triisopropylsilane-H_2_O (95:2.5:2.5) for an hour and a half. Crude peptides were analyzed by ultraperformance liquid chromatography (UPLC)-mass spectrometry (performed with a Waters Acquity ultraperformance liquid chromatograph and a Micromass Quattro micro API mass spectrometer) on an Acquity UPLC BEH C_18_ column (particle size diameter, 1.7 μl; 1.0 by 50 mm) and purified by preparative high-performance liquid chromatography (performed with a Waters 2777 sample manager, a Waters 2545 binary gradient module, and a Waters 2487 dual λ absorbance detector, using a Waters C_18_ Xbridge PreShield RP18 column [19 by 100 mm; particle size diameter, 5 μm]). Samples were lyophilized and quantified with a Qubit (version 2.0) fluorometer (Life Technologies).

### Expression and purification of recombinant monomeric N protein.

Escherichia coli BL21(DE3) bacteria (Novagen, Madison, WI) were transformed with the pET-N^K170A/R185A^ vector, which has been described previously (16). The bacteria were grown at 37°C for 8 h in Luria-Bertani (LB) medium containing kanamycin (50 μg/ml), and then the same volume of LB was added and protein expression was induced by adding 80-μg/ml isopropyl-β-d-thiogalactopyranoside (IPTG) to the medium. The bacteria were incubated for 15 h at 28°C and then harvested by centrifugation. The protein was purified using a C-terminal 6×His tag. Briefly, bacterial pellets were resuspended in lysis buffer (20 mM Tris-HCl, pH 8, 500 mM NaCl, 0.1% Triton X-100, 10 mM imidazole, 1-mg/ml lysozyme) supplemented with cOmplete protease inhibitor cocktail (Roche). After sonication, NaCl was added to obtain a final concentration of 1 M, before centrifugation. The lysates were incubated for 30 min with chelating Sepharose Fast Flow beads charged with Ni^2+^ (GE Healthcare). The beads were then successively washed in washing buffer (20 mM Tris-HCl, pH 8, 1 M NaCl) containing increasing concentrations of imidazole (10, 50, and 100 mM), and the proteins were eluted in the same buffer with 500 mM imidazole. In order to isolate the recombinant monomeric N protein (N^mono^), eluate was loaded onto a Sephacryl S-200 HR 16/30 column (GE Healthcare) and eluted in 20 mM Tris-HCl, pH 8.5, 150 mM NaCl, 5% glycerol.

### CD spectroscopy.

The circular dichroism spectra were acquired on a Chirascan spectropolarimeter. The samples were prepared in 10 mM phosphate buffer, pH 7.5, at a peptide concentration of 50 μM. Data were recorded at 25°C by step scan from 180 nm to 260 nm in a 0.5-mm-pathlength quartz cell using 0.2-nm-wavelength increments, a 1-nm bandwidth, and a response time of 1 s. Each spectrum was an average of three measurements and was subtracted from the buffer baseline. The data were converted to per residue molar ellipticity units (θ; in degrees · square centimeters · decimoles^−1^ · number of residues^−1^) and smoothed using Igor Pro software. Percent helicity was calculated as follows: (100 × CD_222_)/(*C* × *N* × {40,000 × [1 − (2.5/*N*)]}), where CD_222_ is the molar ellipticity (θ) at 222 nm (in millidegrees), *N* is the number of amino acids in the peptide, and *C* is the peptide molar concentration (in moles per liter).

### Fluorescence polarization assay.

The FAM–P(1–40) peptide probe was synthesized by standard SPPS procedures at GenScript. The fluorescence polarization assay was performed in 384-well plates using a SpectraMax Paradigm microplate reader (Molecular Devices) and excitation and emission wavelengths of 485 nm and 535 nm, respectively. The acquisition time was 700 ms, and the read height was 1 mm. Ten microliters of the appropriate peptide inhibitor concentration (serially diluted) in FP buffer (20 mM Tris, 500 mM NaCl, pH 8.5), 10 μl of FAM–P (1-40), and 10 μl of the N^mono^ recombinant protein were added in that order, and the mixture was incubated for 30 min at room temperature in the dark. The final concentration of N^mono^ protein was 2 to 8 μM, and the concentration of the FAM-P probe was 10 nM. The IC_50_ value was calculated using Igor software.

### rHRSV-mCherry inhibition assay.

HEp-2 cells seeded at 5 × 10^4^ cells per well in 96-well plates were infected at a multiplicity of infection (MOI) of 0.2 for 2 h with rHRVS-mCherry diluted in minimum essential medium (MEM) without phenol red and without fetal calf serum (FCS). In parallel, peptides were 2-fold serially diluted in dimethyl sulfoxide (11 dilutions) and then further diluted in MEM without phenol red medium containing 2% SVF. After infection, the medium was replaced by MEM containing the different concentrations of peptides. The plates were incubated 48 h at 37°C, and the mCherry fluorescence was measured using a spectrophotometer (Tecan Infinite M200PRO) with excitation and emission wavelengths of 580 and 620 nm, respectively. The value obtained for noninfected HEp-2 cells was used as a standard for the fluorescence background level, and the value obtained for infected and untreated cells was used to normalize the data. The EC_50_ value was calculated using Igor software. Cytotoxicity assays were done with the CellTiter-Glo luminescent cell viability assay (Promega). Each experiment was performed in duplicate and repeated at least twice.

### Minigenome assay.

BSRT7/5 cells at 90% confluence in 96-well dishes were transfected with a plasmid mixture containing 62.5 ng of pM/Luc, 62.5 ng of pN, 62.5 ng of pP, 31.25 ng of pL, and 15.6 ng of pM2-1, as well as 15.6 ng of pRSV-β-Gal (Promega), to normalize transfection efficiencies (27). Cells were transfected using the Lipofectamine 2000 reagent (Invitrogen, Cergy-Pontoise, France) in Opti-MEM medium (Gibco), as described by the manufacturer. At 6 h posttransfection, the medium was replaced by DMEM without antibiotics containing the stapled peptides at 25 μM. The cells were harvested at 24 h posttransfection and lysed in luciferase lysis buffer (30 mM Tris, pH 7.9, 10 mM MgCl_2_, 1 mM dithiothreitol, 1% Triton X-100, 15% glycerol). The luciferase activities were determined for each cell lysate with an Infinite 200 Pro microplate reader (Tecan, Männedorf, Switzerland) and normalized based on β-galactosidase (β-Gal) expression and on the value obtained for transfected and untreated cells. The transfections were done in triplicate, and each independent transfection experiment was performed three times.

### Mouse infection and treatment.

Female BALB/c mice were purchased from the Centre d’Elevage R. Janvier (Le Genest Saint-Isle, France) and were used at 8 weeks of age. Mice (*n* = 5 per group) were anesthetized with a mixture of ketamine and xylazine (1 mg and 0.2 mg per mouse, respectively) and were treated intranasally (i.n.) with 50 μl of peptide 5a at 215 μM in PBS or PBS for control mice. For the infection assay, the mice were infected i.n. with 50 μl of rHRSV-Luc (2 × 10^6^ PFU/ml) 10 min later. Mouse body weight was measured each day. At 2 days postinfection (dpi), the mice were anesthetized to perform the *in vivo* luminescence measurement and treated i.n. a second time with 50 μl of peptide 5a at 215 μM in PBS. Luminescence measurement was then performed at 4 dpi.

### *In vivo* luminescence measurements.

Mice were anesthetized at 2 and 4 days postinfection (dpi), and bioluminescence was measured 5 min following the i.n. injection of 50 μl of PBS containing 7 mg · kg^−1^ d-luciferin (Sigma). Living Image software (version 4.0; Caliper Life Sciences) was used to measure the luciferase activity. Bioluminescence signals were acquired with an exposure time of 1 min. Digital false-color photon emission images of the mice were generated and show the average radiance (in number of photons per second per square centimeter per steradian). Photons were counted within three different regions of interest (ROI), corresponding to the nose, the lungs, and the whole airway area. Signals are expressed as the total flux normalized to the surface of the ROI (in number of photons per second per square centimeter). The data were analyzed using GraphPad Prism software (version 6). The nonparametric Mann-Whitney test (comparison of two groups, *n* ≥ 4) was used to compare unpaired values (GraphPad Prism software). Significance is indicated in the appropriate figure legends.

### Histological analysis.

The mice were sacrificed at 4 dpi, the chest cavity was opened, and the lungs were perfused intratracheally with 4% paraformaldehyde (PFA) in PBS. The lungs were then removed and immersed in 4% PFA for 12 h before transfer in 70% ethanol. The lungs were embedded in paraffin, and 5-μm sections were cut, stained with hematoxylin-eosin-saffron (HES), and evaluated microscopically. Qualitative histological changes were described and, when applicable, were scored semiquantitatively using a three-point scale ranging from 0 to 2 (0, none; 1, mild; 2, marked), focusing on histological characterization of the lesion (interstitial pneumonia, respiratory epithelial cell apoptosis, and hyperplasia) and inflammation.

### Ethics statement.

The *in vivo* studies were carried out in accordance with INRAE guidelines, which are compliant with the European animal welfare regulation. The protocols were approved by the Animal Care and Use Committee at the Centre de Recherche de Jouy-en-Josas (COMETHEA) under relevant institutional authorization (Ministère de l’éducation nationale, de l’enseignement supérieur et de la recherche; authorization number 201803211701483v2, APAFIS number 14660). All experimental procedures were performed in a biosafety level 2 facility.
